# Impact of warning labels on sugar-sweetened beverages on parental selection: An online experimental study^☆^

**DOI:** 10.1016/j.pmedr.2018.10.016

**Published:** 2018-10-23

**Authors:** Eleni Mantzari, Milica Vasiljevic, Isabelle Turney, Mark Pilling, Theresa Marteau

**Affiliations:** a Behaviour and Health Research Unit, University of Cambridge, UK; b Department of Psychology, University of Cambridge, UK

**Keywords:** SSBs, Sugar Sweetened Beverages, Warning labels, Calorie labels, Graphic warnings, Image-based labels, Energy information, Sugar-sweetened beverages, SSBs

## Abstract

Sugar-sweetened beverages (SSBs) are one of the largest added sugar sources to diets in the UK and USA, particularly among young people. Warning labels, including calorie information labels, could reduce SSB consumption but uncertainty surrounds the labels that are most effective. This study assessed the impact of labels containing (a) each of two image-based warnings and (b) calorie information, singly and together, on SSB selection by parents of 11–16-year-olds living in the UK. Using a 3 (disease image, sugar content image, no image) × 2 (calorie information, no calorie information) between-subjects experimental design, 2002 participants were randomised to see beverages with one of six labels and selected one for their child to consume. The primary outcome was the proportion of participants selecting an SSB. Data were collected in December 2017. Logistic regressions showed SSB selection was lower when labels contained an image-based warning (35%), compared to not having any label (49%) or just calorie information (43.5%). The disease image lowered selection more than the sugar image (32% vs 40.5%). Providing calorie information with the disease image had no additional impact on selection (33%) but enhanced the impact of the sugar image (36%). Image-based warning labels discourage SSB selection by parents for their children. Images depicting health consequences of excess sugar consumption have larger effects than those depicting sugar content. Calorie information does not add to the effect of the former but does to that of the latter. Field studies are needed to assess the impact of SSB warning labels in real-life settings.

## Background

Over a third of children and two thirds of adults in the UK and USA are overweight or obese (NHS Digital, 2018; National Center for Health Statistics, 2017). A major contributor to the development of obesity is the excess intake of added sugars. One of the largest sources of added sugars in people's diet are sugar-sweetened beverages (SSBs) (Public Health England, 2018; Bailey et al., 2018). In the UK, SSBs contribute approximately 15% of total added sugar intake in adults, 10% in children of all ages and as high as 22% in children aged 11–18 years (Public Health England, 2018). In the USA, SSBs contribute approximately 23% of total added sugar intake in adults and young children and as high as 33% in adolescents (Bailey et al., 2018).

Consumption of SSBs is linked to the development of adverse health conditions, including obesity, metabolic syndrome, diabetes and dental decay (Fung et al., 2009; Cohen et al., 2012; Batt et al., 2014; Bachman et al., 2006; Bomback et al., 2010; Larsson et al., 2014; Malik et al., 2010a; Malik et al., 2010b; Malik et al., 2006; Mishra and Mishra, 2011; Schernhammer et al., 2005; Te Morenga et al., 2013; Vartanian et al., 2007). One potential intervention, considered by a number of cities in the USA, including San Francisco, New York and Washington (Samuel, 2016) and highlighted in the UK Government's 2016 Childhood Obesity Strategy (HM Government, 2016), is the use of warning labels on SSBs. Evidence for the impact of warning labels comes from their use on tobacco products, which suggests that both text- and image-based warnings can affect a range of effectiveness outcomes, including cessation-related behaviours (Hammond et al., 2006; Borland and Hill, 1997; Borland, 1997; Hammond et al., 2004; Hammond et al., 2003; Ngo et al., 2018). Image-based labels appear to exert greater effects (Brewer et al., 2016; Noar et al., 2017; Noar et al., 2016; Noar et al., 2015; Evans et al., 2015; Romer et al., 2017; Evans et al., 2018), being more noticeable and eliciting greater negative emotional reactions than text-only labels (Evans et al., 2015; Romer et al., 2017; Hammond et al., 2007; Magnan and Cameron, 2015; Hall et al., 2017). Indeed, the most effective labels are those which include images that elicit a strong negative emotional response (Hammond et al., 2006; Hammond et al., 2004; Hammond et al., 2003; Cho et al., 2017; Nonnemaker et al., 2014).

Comparatively little evidence exists on the impact of warning labels on food or beverages. Findings from the few studies conducted in this area suggest that when used on food products, warning labels can increase dietary self-control (Rosenblatt et al., 2018), reduce appetite, decrease intentions to consume and purchase labelled products (Ares et al., 2018; David et al., 2018) and promote healthier food purchasing (Mhurchu et al., 2018; Neal et al., 2017). Similarly, when used on alcoholic beverages, warning labels can slow consumption (Stafford and Salmon, 2017) and reduce intentions to drink (Wigg and Stafford, 2016). Their use on SSBs also shows promise. The findings from a recent simulation study suggest that implementing warning labels, whether text or image-based, on SSBs across all retailers could reduce obesity prevalence among adolescents (Lee et al., 2017). Text-based labels can improve understanding of the health harms associated with SSBs overconsumption and may reduce the selection of such drinks (Bollard et al., 2016; Gray et al., 2011; Vanepps and Roberto, 2016), including by parents choosing beverages for their children (Roberto et al., 2016). Consistent with prior research on the use of warning labels on tobacco products, image-based labels on SSBs, including labels that illustrate the health consequences of excess sugar consumption (Bollard et al., 2016) and those illustrating sugar content (Adams et al., 2014), appear superior than text-based labels, having been shown more effective at reducing intentions to purchase SSBs and preferences for SSBs (Bollard et al., 2016; Adams et al., 2014). Further research, however, is needed to elucidate the types of images that could be most effective.

Recent evidence suggests that compared to not having any labels, labels with images illustrating sugar content and those with images illustrating the health consequences of excess sugar consumption both reduce SSB selection in young adults (Billich et al., 2018). Although the latter appears to have a greater effect, the two types of images have not been directly compared against each other to determine whether they exert differential impacts. Based on evidence from the use of warning labels on tobacco products, it is predicted that the most effective SSB labels will be those that use images that elicit the most negative reactions. The impact of each image type on negative emotional arousal and whether this mediates observed effects on selection is currently unknown. Images that elicit strong negative reactions might be considered less acceptable by consumers and policy makers. As an intervention's acceptability could have implications for its implementation (Diepeveen et al., 2013), identifying images that are both acceptable and effective is important. There is a need, therefore, to also address the lack of evidence regarding the impact of different types of image-based warnings on acceptability.

Another type of label that has been recommended by the Word Health Organisation (WHO, 2014) for facilitating healthier purchasing and consumption choices, are labels comprising nutritional information, including energy content (calories). Although the evidence regarding the impact of labelling single food or drink options, such as soft drinks, with nutritional information is inconclusive (Crockett et al., 2018), labelling comprising energy information on menus or adjacent to products has the potential to change people's choice at point of selection and consumption (Crockett et al., 2018). This is consistent with findings showing that calorie information may reduce selection and consumption specifically of SSBs (Bleich et al., 2014; Bleich et al., 2012). Taken together, these findings highlight both the promising use of labels on calorie information and the need for further research in this area. The additive effect of combining calorie information with warnings on labels also requires exploration.

The present research aims to identify the labels with the greatest potential to affect SSB selection and thereby consumption. The primary aim is to assess the impact on SSB selection of: i) each of two image-based warning labels, one depicting an adverse health consequence of excess SSB consumption, and one depicting sugar content; and ii) calorie information labels. Secondary aims are to assess (a) the impact of each label on perceptions of SSBs and acceptability of using the different labels on SSBs; and (b) the mediating role of negative emotional arousal on the impact of labels on SSB selection.

## 1 Methods

### 1.1 Design

The study was conducted online with the Qualtrics software, using a 3 (image-based warning label) × 2 (calorie information label) between-subjects factorial design. Participants were randomised to one of six possible groups (Box 1).

Box 1 **Study design⁎.**

**Table:** 

	Calorie information	
Warning Image	Absent	Present
No image	Group 1:	Group 2:
Health consequence of excess sugar consumption (Disease)	Group 3:	Group 4:
Sugar content	Group 5:	Group 6:

⁎ Images are for illustrative purposes only. Images of a range of branded drinks were used.

Alt-text: Box 1

### 1.2 Participants

**Fig. 1:**
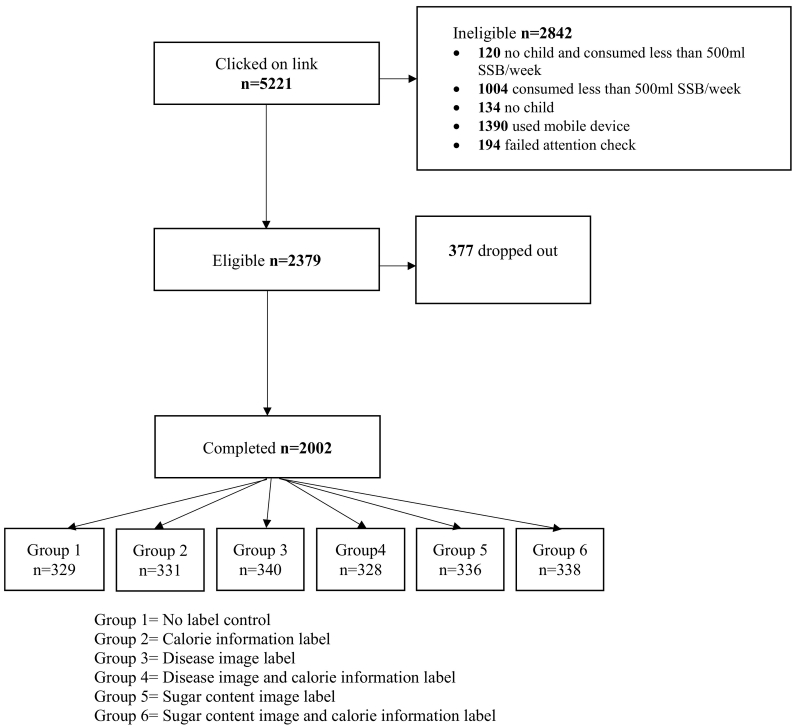
Flow of participants through study.

Participants were 2002 parents of children aged 11–16 years living in the UK, with a total household SSB consumption of at least 500 ml each week, recruited via a market research agency. Fig. 1 shows the flow of participants through the study and Table 1 their characteristics across groups.

**Table 1:** Characteristics of participants in each group (n (%))^a^.

	Group 1 control *n* = 329	Group 2 calories *n* = 331	Group 3 disease *n* = 340	Group 4 disease & calories *n* = 328	Group 5 sugar *n* = 336	Group 6 sugar & calories *n* = 338
Household consumption (volume per week)^b^						
500 ml-1 l	82 (25%)	78 (24%)	78 (23%)	75 (23%)	23% (77)	72 (21%)
1 l–1.5 l	72 (22%)	78 (24%)	63 (19%)	57 (17%)	21% (69)	46 (14%)
1.5 l–2 l	57 (17%)	39 (12%)	66 (19%)	64 (19%)	16% (52)	66 (19%)
2 l–2.5 l	37 (11%)	36 (11%)	43 (13%)	39 (12%)	13% (44)	49 (14%)
2.5 l–3 l	24 (7%)	27 (8%)	28 (8%)	23 (7%)	9% (30)	40 (13%)
>3 l	57 (17%)	73 (22%)	62 (8%)	70 (21%)	19% (64)	65 (19%)

Household's preferred drink^c^						
Cola	151 (46%)	153 (46%)	168 (49%)	149 (45%)	162 (48%)	170 (50%)
Fizzy orange	31 (9%)	43 (13%)	33 (10%)	32 (10%)	30 (9%)	34 (10%)
Fizzy lime/lemon	24 (7%)	16 (5%)	17 (5%)	28 (8%)	29 (9%)	16 (5%)
Squash	88 (27%)	80 (24%)	77 (23%)	80 (24%)	85 (25%)	81 (24%)
Ice Tea	12 (4%)	9 (3%)	5 (2%)	8 (2%)	7 (2%)	8 (2%)
Energy drink	8 (2%)	12 (4%)	9 (3%)	11 (3%)	5 (2%)	6 (2%)
Sports drink	15 (5%)	18 (5%)	31 (9%)	20 (6%)	18 (5%)	23 (7%)
Age (sd)	44.6 (8.1)	43.5 (8.8)	42.9 (8.5)	43.9 (8.8)	44.1 (8.7)	44.1 (9.0)

Gender						
Male	173 (53%)	182 (55%)	171 (50%)	174 (53%)	170 (51%)	169 (50%)
Female	156 (47%)	148 (45%)	169 (50%)	153 (47%)	166 (49%)	170 (50%)

Ethnicity						
White	292 (89%)	303 (92%)	305 (89%)	298 (90%)	302 (90%)	316 (93%)
Mixed	10 (3%)	4 (1%)	7 (2%)	9 (3%)	10 (3%)	8 (2%)
Asian	17 (5%)	17 (5%)	19 (6%)	13 (4%)	14 (4%)	8 (2%)
Black	10 (3%)	5 (2%5)	9 (3%)	8 (2%)	8 (2%)	4 (1.5%)
Prefer not to say		2 (1%)			2 (1%)	2 (0.5%)

Education						
Low	113 (34%)	96 (29%)	102 (30%)	108 (33%)	101 (30%)	119 (35%)
Higher	216 (66%)	235 (71%)	233 (69%)	215 (65%)	235 (70%)	217 (64%)
Prefer not to say			5 (1%)	2 (2%)		2 (1%)

a There were no statistically significant differences between groups in any of the participant characteristics. Randomisation to experimental groups was therefore successful.

b Participants were asked: “What amount of sugary drinks (e.g. non-diet drinks: regular cola, non-diet squash, sports drinks etc) in total does your household consume in an average week?”

c Participants were asked: “What type of sugary drink do you most often drink in your household? Please choose from the list below”.

Based on previous research (Roberto et al., 2016), the expected difference in the proportion of parents selecting an SSB between those allocated to a control group and those allocated to a warning label group was 12.9% (control = 53.3% vs warning labels = 40.4%). It was possible to recruit a sample size of approximately 2000 (333 participants per group). This allowed a proportion to be estimated with a precision (i.e. a 2-sided 95%CI) of a least ±0.054, thus differences of at least 10.8% between proportions to be detected, and be more than sufficient to accommodate logistic regression analyses (Peduzzi et al., 1996).

### 1.3 Interventions

(a) Image-based warnings

The labels used in the study were chosen based on the results of a pilot study, which aimed to identify the images eliciting the highest levels of negative emotional arousal. The pilot study was conducted online using a within-subjects design, in which 1002 parents viewed 11 different image-based warning labels presented on cola bottles in counterbalanced order. Seven labels included images of the health consequences of excess sugar consumption (disease image labels) and four labels included images illustrating sugar content (sugar content image labels). Each image was followed by questions to assess negative emotional arousal, rated on a 1–7 scale. Disease image labels resulted in overall significantly higher levels of negative emotional arousal (disease: *M* = 5.00, *SD* = 1.42; sugar content: *M* = 3.96, *SD* = 1.70), *F*(4.31, 4313) = 320.93, *p* < .001). The images eliciting the highest levels of negative emotional arousal within each label category were used in the main study:

i) *Disease image label*: an image of rotting teeth alongside the caption “Excess sugar intake causes dental decay” (*M* = 5.61 *SD* = 1.50);
ii) *Sugar content image label:* an image depicting a teaspoon of sugar accompanied by the number of teaspoons contained in the SSB, alongside the caption “There are x number of teaspoons of sugar in this bottle” (*M* = 4.14, *SD* = 1.89).

More information on the methods, including the images used, and results of the pilot study can be found in the Supplementary data (Appendix A).

(b) Calorie information

This comprised the number of calories contained in each drink. The number was shown using numerals in black font against a white background and placed at the front right bottom corner of the container. The volume of each drink was kept constant (500 ml). In groups containing calorie information on the label, all drinks, including both SSBs and non-SSBs, showed calorie information per bottle.

See Box 1 for examples of the labels used in the study.

### 1.4 Outcomes

#### 1.4.1 Primary

• Proportion of participants selecting an SSB in the vending machine task (see Procedure)

#### 1.4.2 Secondary

Perceptions of SSBs (See Appendix B, Table S5, Supplementary data for relevant questions)

• Negative emotional arousal
• Perceived risks of drinking SSBs
• Acceptability of introducing image-based label and/or calorie information label on SSBs

### 1.5 Procedure

Ethical approval for this study was granted by the Cambridge Psychology Research Ethics Committee (PRE.2017.049). After consenting to participate, participants were requested to complete screening questions. Eligible participants were subsequently asked questions regarding their demographic characteristics (gender, age, ethnicity and education level) and preferred type of SSB. In order to ensure that images were clearly visible, the study had to be completed on a computer-sized screen. Inattentive participants were screened out via an attention check question and sampling continued until the quota was filled. All participants who successfully completed the study were debriefed and reimbursed for their participation. Data were collected in December 2017.

#### 1.5.1 Vending machine task

After completing the screening and demographic questions, participants were randomised to one of six groups (Box 1). All participants simultaneously viewed images of 18 drinks presented in random order (12 SSBs and 6 non-SSBs). Participants were asked to imagine they were looking at drinks in a vending machine and were about to select one for their child. Depending on their allocated group, SSBs either had no warning label, or one of two warning labels and all drinks had either no calorie information or a calorie information label.

#### 1.5.2 Perceptions and acceptability

Following completion of the ‘vending machine task’, participants viewed an image of a cola bottle with or without a label depending on their allocated group, and were asked to answer questions relating to their perceptions of SSBs and label acceptability (See Supplementary data – Appendix B Table S5). Participants could not proceed without answering all questions.

### 1.6 Statistical analyses

Descriptive proportions of SSB selection with normal approximation 95%CI were calculated using SPSSv25 (Corp, 2017). Logistic regressions were performed to assess the odds of selecting an SSB in each group. Each experimental group was compared against each of the other groups, by varying the reference group and repeating the analysis (i.e. in total six logistic regressions were performed).

For continuous outcomes, normality was assessed through inspection of QQ plots and the randomness of residuals. Linear regressions were conducted for outcomes where minimal deviation from the QQ plot line was observed (negative emotional arousal). Where some deviations from normality were observed, robust regressions to gain parameter variances were performed (acceptability, perceived risks). Exploration of the histogram for perceived health risk scores revealed an edge peak distribution with high frequency in those scoring 7/7. Adding those individuals as a separate predictor improved the model. To compare the outcomes in each group against each of the other groups, linear/robust regressions were repeated, by varying the reference group.

Mediation analyses were conducted to assess the mediating role of negative emotional arousal on SSB selection.

## 2 Results

Descriptive information regarding the outcomes according to each group can be seen in Table 2.

**Table 2:** Primary (percentages (95%CI)) and secondary outcomes (mean (sd)) according to group.

Outcome	Group 1 control n = 329	Group 2 calories n = 331	Group 3 disease n = 340	Group 4 disease & calories n = 328	Group 5 sugar n = 336	Group 6 sugar & calories n = 338
PRIMARY						
Proportion choosing SSB^a^	49.2% (47.0%–51.4%)	43.5% (41.3%–45.7%)	32.4% (30.3%–34.4%)	32.6% (30.5%–34.6%)	40.5% (38.3%–42.6%)	35.8% (33.7%–37.9%)
SECONDARY						
Negative emotional arousal^b^	2.4 (1.7)	2.9 (1.7)	5.0 (1.6)	4.7 (1.8)	4.6 (1.8)	4.5 (1.8)
Perceived health risks^c^	5.1 (1.1)	5.2 (1.1)	5.3 (1.1)	5.3 (1.2)	5.5 (1.2)	5.4 (1.2)
Weight gain	5.1 (1.5)	5.1 (1.5)	5.2 (1.5)	5.2 (1.6)	5.4 (1.6)	5.4 (1.5)
Develop heart disease	4.7 (1.4)	4.7 (1.5)	4.9 (1.4)	4.9 (1.5)	5.3 (1.4)	5.1 (1.5)
Develop diabetes	5.0 (1.4)	5.2 (1.4)	5.4 (1.4)	5.4 (1.5)	5.6 (1.4)	5.5 (1.4)
Lead healthy life (reverse scored)	5.6 (1.6)	5.5 (1.6)	5.6 (1.7)	5.7 (1.8)	5.8 (1.7)	5.7 (1.8)
Acceptability	3.6 (1.9)	5.7 (1.5)	5.2 (1.7)	5.3 (1.7)	5.9 (1.4)	6.2 (1.2)

a The choice was made between 12 SSBs (Coca Cola; Sprite; IronBru; Lucozade; PowerAde; Lipton Ice Tea Peach; Ribena Blackcurrant; Yazoo Milkshake Banana; Fanta Orange; Fanta Twist; Dr. Pepper; Oasis citrus punch) and 6 non-SSBs (Volvic water; Tropicana orange juice; Lucozade lite; 7up Free; Diet Coke; Ribena light).

b Aggregate measure of four items measuring fear, worry, disgust, discomfort; Cronbach's α = 0.95.

c Aggregate measure of the four listed items; Cronbach's α =0.75.

### 2.1 Impact on SSB selection

Compared to the control group use of all image-based warning labels decreased the odds of selecting an SSB (disease image: OR = 0.493; 95%CI = 0.360, 0.675; disease image with calorie information; OR = 0.499; 95%CI = 0.3604, 0.685; sugar content image: OR = 0.701, 95%CI = 0.516, 0.953; sugar content image with calorie information: OR = 0.575; 95%CI = 0.422, 0.784), There was no significant difference in SSB selection between the control group and calorie information group (OR = 0.794, 95%CI = 0.584, 1.079). Compared to calorie information alone, use of all labels, apart from the sugar content label, decreased SSB selection. The disease image label was superior at decreasing the odds of SSB selection compared to the sugar content image label (OR = 1.422, 95%CI = 1.038, 1.948) but not the sugar content plus calorie information label. Adding calorie information to the disease image did not have an additive effect. Adding calorie information to the sugar content image was not more effective than the sugar content label alone but was more effective than using calorie information alone (OR = 0.724, 95%CI = 0.531, 0.988). (Table 3).

**Table 3:** ORs (95% CI) of choosing an SSB in each condition against all others.

Reference group^a^	Control	Calorie information	Disease image label	Disease image & calorie information label	Sugar content image label	Sugar content image & calorie information label
Control	–	0.794 (0.584–1.079)	0.493⁎⁎⁎ (0.360 –0.675)	0.499⁎⁎⁎ (0.364–0.685)	0.701⁎ (0.516–0.953)	0.575⁎⁎⁎ (0.422–0.784)
Calorie information	1.260 (0.927–1.712)	–	0.621⁎⁎ (0.454–0.851)	0.629⁎⁎ (0.459–0.863)	0.883 (0.649–1.201)	0.724⁎ (0.531–0.988)
Disease image label	2.028⁎⁎⁎ (1.482–2.775)	1.610⁎⁎ (1.176–2.205)	–	1.012 (0.732–1.400)	1.422⁎ (1.038–1.948)	1.166 (0.848–1.602)
Disease image & calorie information label	2.004⁎⁎⁎ (1.460–2.749)	1.590⁎⁎ (1.158–2.184)	0.988 (0.715–1.366)	–	1.404⁎ (1.023–1.029)	1.152 (0.836–1.587)
Sugar content image label	1.427⁎ (1.050–1.939)	1.132 (0.832–1.540)	0.703⁎ (0.513–0.964)	0.712⁎ (0.518–0.978)	–	0.820 (0.601–1.119)
Sugar content image & calorie information label	1.740⁎ (1.276–2.572)	1.381⁎⁎⁎ (1.012–1.885)	0.858 (0.624–1.179)	0.868 (0.630–1.196)	1.220 (0.893–1.605)	–

⁎ Significant at the <0.05 level.

⁎⁎ Significant at the <0.005 level.

⁎⁎⁎ Significant at the <0.001 level.

a Reference group: first row: Control; second row: Calorie information; third row: Disease image label; fourth row: Disease image & Calorie information label; fifth row: Sugar content image label; sixth row: Sugar content image & Calorie information label.

### 2.2 Impact on perceptions of SSBs

#### 2.2.1 Negative emotional arousal

Compared to the control group, all labels significantly increased levels of negative emotional arousal (calorie information: OR = 0.516, 95%CI = 0.252, 0.779; disease image: OR = 2.56, 95%CI = 2.305, 2.829; disease image with calorie information: OR = 2.273, 95%CI = 2.008, 2.537; sugar content image: OR = 2.202, 95%CI = 1.939, 2.465; sugar content image with calorie information: OR = 2.040, 95%CI = 1.777, 2.302) (Table 2; Table S6, Appendix B, Supplementary data). Both types of image-based warning labels with and without calorie information elicited greater negative emotional arousal compared to calorie information alone (disease image: OR = 2.051, 95%CI = 1.790, 2.313; disease image with calorie information: OR = 1.757, 95%CI = 1.493, 2.021; sugar content image: OR = 1.687, 95%CI = 1.424, 1.949; sugar content image with calorie information: OR = 1.524; 95%CI = 1.262, 1.786). The disease image label alone elicited greater negative emotional arousal compared to all other labels (disease image with calorie information: OR = −0.293, 95%CI = −0.557, −0.032; sugar content image: OR = −0.365, 95%CI = −0.625, −0.104; sugar content image with calorie information: OR = −0.525; 95%CI = −0.788, −0.267). Adding calorie information to the disease image resulted in less negative emotional arousal than the disease image alone (OR = 0.294; 95%CI = 0.032, 0.557) but not compared to both sugar content image labels. Adding calorie information to the sugar content image had no significant impact on negative emotional arousal (Table 2; Table S6, Appendix B, Supplementary data).

#### 2.2.2 Perceived risks of drinking SSBs

Compared to both the control (disease image: OR = 0.142; 95%CI = 0.000, 0.283; sugar content image: OR = 0.173, 95%CI = 0.030, 0.317; sugar content image with calorie information: OR = 0.178, 95%CI = 0.034, 0.321) and calorie information label (disease image: OR = 0.179, 95%CI = 0.037, 0.320; sugar content image: OR = 2.11, 95%CI = 0.966, 2.253; sugar content image with calorie information: OR = 0.215, 95%CI = 0.073, 0.356), the disease image label and both sugar content image labels increased the perceived risks of drinking SSBs. Calorie information alone and the disease image with calorie information label did not have any effect compared to all other groups (Table S6, Appendix B, Supplementary data).

#### 2.2.3 Acceptability of labels

Compared to the control group, all labels resulted in significantly higher levels of acceptability (calorie information: OR = 2.407, 95%CI = 2.154, 2.659; disease image: OR = 2.021, 95%CI = 1.769, 2.272; disease image with calorie information: OR = 2.227, 95%CI = 1.972, 2.481; sugar content image: OR = 2.647, 95%CI = 2.394, 2.899; sugar content image with calorie information: OR = 2.849, 95%CI = 2.596, 3.101). The disease image label was less acceptable than calorie information alone (OR = 0.386, 95%CI = 0.134, 0.637) and both disease labels with and without calorie information were less acceptable than the sugar content image label (disease image: OR = 0.626, 95%CI = 0.374, 0.877; disease image with calorie information: OR = 0.420, 95%CI = 0.672, 0.167) and the sugar content image label with calorie information (disease image: OR = 0.828, 95%CI = 0.576, 1.079; disease image with calorie information: OR = 0.651, 95%CI = 0.399, 0.902). The sugar content image alone was not more acceptable than the calorie information label but the sugar content image with calorie information was more acceptable than calorie information alone (OR = −0.442; 95%CI = −0.693, −0.190) (Table S6, Appendix B, Supplementary data).

### 2.3 Mediating effect of negative emotional arousal

Negative emotional arousal mediated the effect on SSB selection of the disease image label when compared to both the control (*Z*_Sobel_ = −2.25, *p* = .024) or calorie information label (*Z*_Sobel_ = −2.57, *p* = .01). It did not mediate the effect on SSB selection of the disease image with calorie information, nor labels with sugar content images (both with and without calorie information) when compared to the control or calorie information labels. No mediating effect was found when contrasting the impact of labels containing calorie information alone when compared to the control.

## 3 Discussion

Addition of an image-based warning label on SSB packaging reduced selection of SSBs by parents choosing a beverage for their children, compared to not having any label or just having calorie information. The disease image label was superior at reducing SSB selection when compared to the sugar-content label. Addition of calorie information to the disease image did not have any additive effect on reducing SSB selection. The sugar content image label was not better than calorie information alone at reducing SSB selection but addition of calorie information improved its effectiveness.

The findings from this study are consistent with evidence regarding the efficacy of aversive images for discouraging unhealthy dietary choices (Hollands and Marteau, 2016; Hollands et al., 2011), as well as prior findings of the few studies on the use of warning labels on SSBs (Lee et al., 2017; Bollard et al., 2016; Gray et al., 2011; Vanepps and Roberto, 2016; Roberto et al., 2016). The effect size of warning labels overall on SSB selection is similar to that obtained in the previous research based on which this study was powered (Roberto et al., 2016). Research on the use of warning labels on tobacco products suggests that the most effective labels are those which include images that elicit a strong negative emotional response (Hammond et al., 2006; Hammond et al., 2004; Evans et al., 2015; Romer et al., 2017; Hammond et al., 2007; Magnan and Cameron, 2015; Hall et al., 2017; Cho et al., 2017; Nonnemaker et al., 2014). Consistent with this, in the present study, the disease image label, was more effective than the sugar content image label at reducing SSB selection, an effect mediated by elicitation of higher levels of negative emotional arousal. One explanation for this is that the threat was more explicit with the disease image label. Indeed, this label also elicited the highest levels of perceived risk of drinking SSBs. With the sugar content image label, individuals needed to process the sugar content information and link it with their knowledge of the adverse health consequences of high sugar consumption. It is possible that some individuals may not have fully comprehended the extent of the consequences of sugar consumption, thus perceiving the high sugar content of SSBs as less of a threat compared to directly being told of the negative health consequences. This is consistent with the present findings showing that although the sugar content image labels elicited higher levels of negative emotional arousal compared to not having any label or calorie information alone, this did not mediate their effect on SSB selection. The addition of calorie information improved the effectiveness of the sugar content image label perhaps by providing an additional cue for the ‘unhealthiness’ of the product, one that is generally well understood (Van Kleef et al., 2008).

The findings further suggest that apart from improving the impact of sugar content image labels, calorie information alone did not influence SSB choice. This is consistent with the findings of a recent study, which found that calorie labels did not affect SSB selection (Roberto et al., 2016) and implies that current efforts to place nutritional information on beverages may have little influence. Calorie information might even have unintended effects when used in combination with some warning labels. In the present study, when added to the disease image, calorie information resulted in less negative emotional arousal and reduced perceived risks of drinking SSBs. Visual distractions, including perceptual and cognitive tasks, have been shown to interfere with affective reactions to aversive images (Blair et al., 2007; Hajcak et al., 2007; McRae et al., 2010; Schupp et al., 2007; Van Dillen et al., 2009). It is possible, therefore, that in the present study, the calorie information might have distracted participants, thus preventing them from developing an emotional reaction towards the warning label and processing the associated risks.

Although more effective at reducing SSB selection by parents, the disease image label was considered less acceptable than the sugar content image and calorie information labels in the present study. This is consistent with evidence showing that the more effective interventions are often the least acceptable (Diepeveen et al., 2013). The lower levels of acceptability were most likely related to the graphic nature of the image, which was chosen to elicit high levels of negative emotional arousal, including disgust and discomfort. As successful intervention implementation depends in part on its acceptability by the public and policy makers (Diepeveen et al., 2013), identifying labels that are both effective and acceptable is important. In the present study the most acceptable label involved the sugar content image in combination with calorie information. The acceptability of using disease image labels on SSBs, such as the one used in the present study, should be explored further with more extensive populations, preferably in more realistic settings. It is worth noting, however, that although the disease image label was rated as less acceptable than other labels, it was considered more acceptable than not having any label. This mirrors recent findings showing support for a policy to place warning labels on SSBs (Vanepps and Roberto, 2016; Roberto et al., 2016) and is consistent with evidence that labelling intervention for SSBs are typically better supported by the public, at least compared to more intrusive policies such as taxation (Gollust et al., 2014).

The research presented herein builds on the limited evidence on the use of warning labels on SSBs. It is the first and largest to our knowledge to directly compare the impact of different types of image-based labels on selection of SSBs, their additive effect with calorie information, as well as their impact on negative emotional arousal, thus providing valuable insight into the types of images and labels that might be most effective for reducing SSB consumption. It also contributes to the limited evidence regarding the public acceptability of warning labels on SSBs and is the first study to assess such support in a UK-based population sample.

The use of warning labels on SSBs is considered by UK and US governments as a possible intervention to tackle excess sugar consumption. The findings from this study have the potential to inform policy discussions on the design, effectiveness and acceptability of such labels. The study, however, has some limitations that compromise the breadth of the conclusions that can be drawn. First, as it was conducted online using a hypothetical scenario, it remains unclear whether similar results would be obtained in real-world settings. Furthermore, although the study sample was large, it consisted primarily of highly educated, White parents making a drink selection for their children. It remains unclear whether the findings are applicable to populations with varying demographics, including those of lower socioeconomic status. Further research is needed to examine the generalisability of the findings to more naturalistic settings, using more varied populations, and assessing the impact of warning labels on actual purchasing and consumption-related behaviours.

In conclusion, image-based warning labels, especially those illustrating the health consequences of excess sugar consumption, have the potential to reduce the selection of SSBs by parents for their children. Although labels conveying calorie information do not appear to influence choices when presented on their own, or when added to disease image labels, they can increase the impact of image-based warning labels depicting sugar content. Further research in the form of field studies is needed to assess the impact of labels containing image-based warnings and calorie information on SSB selection and consumption in real-life settings.

## Funding source

This work was supported by a grant from the National Institute for Health Research, Policy Research Programme (Policy Research Unit in Behaviour and Health [PR-UN-0409-10109]).

## Contributors' statement

Dr. Mantzari, contributed to conceptualising and designing the study, was responsible for preparation of the study materials and collecting the data, contributed to the analysis and interpretation of the data and prepared the initial manuscript. Dr. Vasiljevic contributed to conceptualising and designing the study, was responsible for preparation of the study materials and collecting the data, contributed to the analysis and interpretation of the data and reviewed and revised the manuscript. Ms. Turney contributed to preparation of the study materials, collection and analysis of the data and reviewed and revised the manuscript. Dr. Pilling was responsible for analysis of the data and reviewed and revised the manuscript. Prof Marteau contributed to conceptualising and designing the study and reviewed and revised the manuscript. All authors approved the final manuscript as submitted and agree to be accountable for all aspects of the work.

## Financial disclosure

The authors have no financial relationships relevant to this article to disclose. This report is independent research commissioned and funded by the National Institute for Health Research Policy Research Programme. The views expressed in this publication are those of the author(s) and not necessarily those of the NHS, the National Institute for Health Research, the Department of Health and Social Care or its arm's length bodies, and other Government Departments.

## Conflicts of interest

The authors have no conflicts of interest relevant to this article to disclose.

## Supplementary data

Appendix A - Pilot study; Appendix B - Main study: Tables S5 and S6. Image 7
