# Supplementary material for: Impact of warning labels on sugar-sweetened beverages on parental selection: An online experimental study
Source: Prev Med Rep. 2018 Oct 23;12:259–67. doi: 10.1016/j.pmedr.2018.10.016 (PMC6215029; doi:10.1016/j.pmedr.2018.10.016)
Supplement: Supplementary file 1 — Appendix A - Pilot study; Appendix B - Main study: Tables S5 and S6. [file mmc1.docx]

**Supplementary data**

**Appendix A – Pilot study**

**Aim**

To identify the images within each warning label category (i.e. labels illustrating the health consequences of excess sugar consumption and labels illustrating drink sugar content) for use in the main study.

**Methods**

Design

Online study conducted using the Qualtrics software ([www.qualtrics.com)](http://www.qualtrics.com)), with a within-subjects design, in which participants viewed 11 different image-based warning labels in random order.

Participants

Participants were 1002 parents of children aged 11-16 years, with a total household consumption of at least 500ml of SSBs per week. They were recruited through a market research agency (Survey Sampling International). In order to ensure that images were clearly visible the survey had to be completed on a computer size screen. Use of smart phones and tablets was not permitted. The flow of participants through the study is presented in Figure S1. Their demographics can be found in Table S2.

Label development

Development of the disease image labels was based on reviewing the literature on the health consequences of excess sugar consumption. Possible ways of illustrating various conditions and diseases were discussed among a panel of experts. The specific images used in this study were chosen based on a pre-pilot study, which was completed by 30 students at the University of Cambridge. Selection was based on rating of how powerful and worrying the images were. The selected labels and their titles can be seen in Figure S1.

To choose the sugar content images, brainstorming sessions were initially held to generate ideas on possible ways to visually illustrate sugar content and nine possible labels were generated. Possibilities were then discussed during meetings with experts in the field of behaviour change and public health, and four images considered most appropriate and potentially effective at deterring consumption and purchase of SSBs were selected. The selected sugar content images and their titles can be seen in Figure S2.

The specific text used alongside the images was reviewed and approved by members of the research community in the field of behaviour change and health.

Primary outcome

Negative emotional arousal, assessed using a four-question measure previously used to assess the impact of warning labels on cigarette packages (Kees, Burton, Andrews, & Kozup, 2006)^[[1]](#footnote-1)^ (Table S2).

Procedure

Ethical approval for the study was granted by the University of Cambridge’s Department of Psychology Ethics Committee (2016/17-12). Following consent and completion of screening and demographic questions, participants viewed 11 different image-based warning labels presented on cola bottles in counterbalanced order. Seven labels included images of the health consequences of excess sugar consumption (disease labels) (Figure S1) and four labels included images illustrates beverage sugar content (Figure S2). Examples of the images that participants viewed can be seen in Figure S3. Each cola image was followed by questions to assess negative emotional arousal (Table S2).

Sample size calculations

The sample size was chosen based on resources and for pragmatic reasons but ensured adequate power, being able to detect a very small effect (f=0.05) with more than 95% power at the 5% significance level.

Statistical analysis

Paired sample *t*-tests were used to assess differences in negative emotional arousal between disease image labels overall and sugar content labels overall. To test for differences between individual labels within each label category, one-way repeated measures ANOVAs with a Bonferroni correction were used.

**Figure S1**: Images illustrating the health consequences of excess sugar consumption


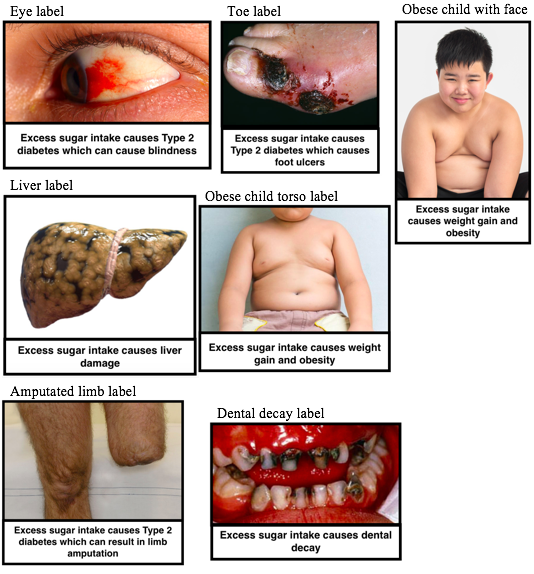


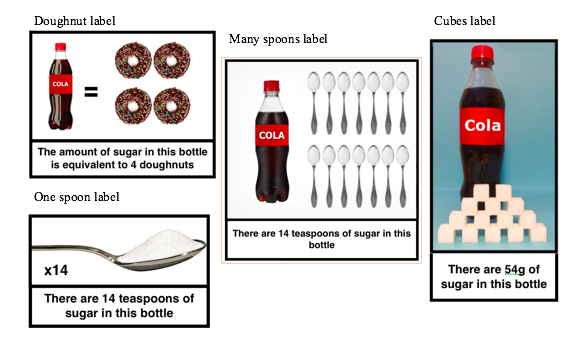
**Figure S2**: Images illustrating beverage sugar content

**Figure S3:** Examples of images viewed by participants (images were viewed one at a time)


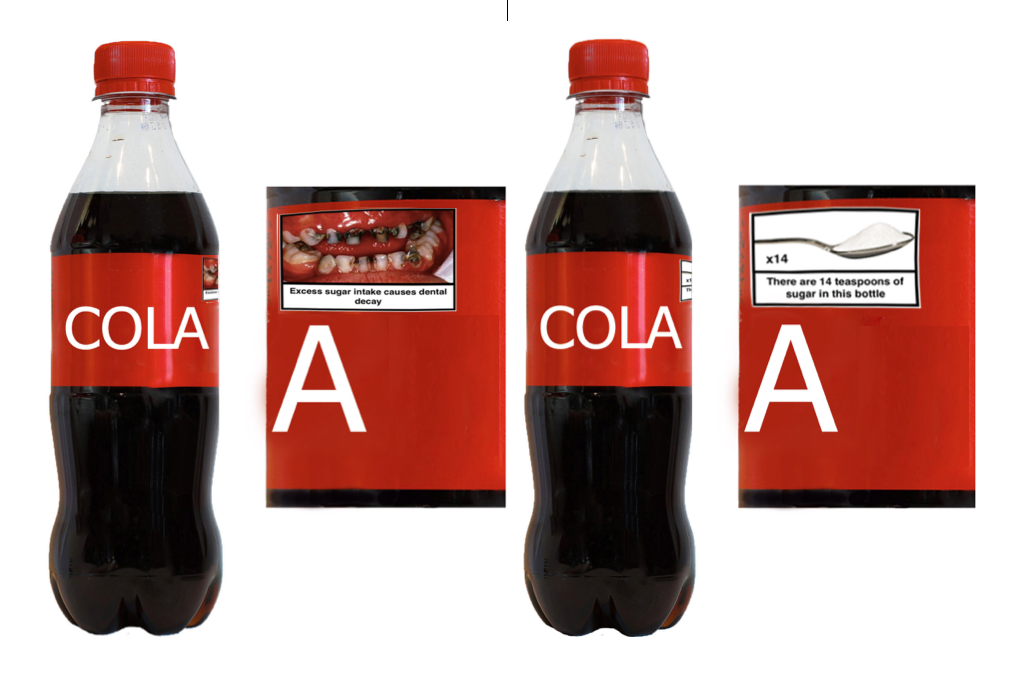


**Table S1: Demographics of Study 1 Participants**

| **Gender**  Male  Female | 45%  55% |
| --- | --- |
| **Ethnicity**  White  Black  Asian  Mixed | 90%  4%  4%  2% |
| **Education Level**  None  Up to 4 GCSEs or equivalent  5 or more or 1 A-level or equivalent  2 or more A-levels or equivalent  Undergraduate degree  Post-Graduate degree  Other vocational/work-related qualifications | 3%  14%  19%  21%  26%  14%  2% |

**Table S2: Outcome measure**

| Measure | Question | Scale |
| --- | --- | --- |
| Negative Emotional Arousal* | How afraid does this image make you feel? | 1 = not at all; 7 = very |
|  | How worried does this image make you feel? | 1 = not at all; 7 = very |
|  | How uncomfortable does this image make you feel? | 1 = not at all; 7 = very |

*Aggregate score for each label was calculated. Cronbach’s alpha demonstrated very high consistency between the four questions (range of *α* = 0.94-0.96).

**Results**

Compared to the sugar content labels, disease labels resulted in significantly higher levels of negative emotional arousal (*F*(4.309, 4313) = 320.933, *p* < .001). There were significant differences in levels of negative emotional arousal elicited by the different disease labels *F*(4.30, 43139) = 320.933, *p* < .001 as well as the different sugar content labels *F*(2.877, 2979) = 62.81, *p* < .001. Post-hoc pairwise comparisons revealed that the majority of the differences between the individual labels were significant. The images eliciting the highest levels of negative emotional arousal within each label category were: dental decay (disease label) (*M* = 5.61 *SD* = 1.50) and teaspoon of sugar accompanied by the number of teaspoons contained in the drink (sugar content) (*M* = 4.14, *SD* = 1.89).

**Figure S4: Flow of participants through Study 1**

Ineligible **n=2842**

- **81** no child and consumed less than 500ml SSB/week
- **414** consumed less than 250ml SSB/week
- **134** no child
- **280** used mobile device
- **81** failed attention check

Completed **n=1002**

Eligible **n=2379**

**198** dropped out

Clicked on link

**n=2190**

**Table S3: Mean (standard deviations) Negative Emotional Arousal scores for each disease image label**

| Label | Mean | Standard deviation |
| --- | --- | --- |
| Dental decay | 5.61 | 1.50 |
| Toe | 5.49 | 1.57 |
| Amputated ^a^ | 5.24 | 1.68 |
| Liver ^a^ | 5.16 | 1.68 |
| Eye | 4.94 | 1.72 |
| Obese child torso ^b^ | 4.27 | 1.76 |
| Obese child with face ^b^ | 4.27 | 1.79 |

Values with the same letters are not significantly different at the p<.05 level

**Table S4: Mean (standard deviations) Negative Emotional Arousal scores for each sugar content image label**

| Label | Mean | Standard deviation |
| --- | --- | --- |
| One Spoon ^a^ | 4.14 | 1.89 |
| Many spoons ^a^ | 4.12 | 1.86 |
| Cubes | 3.92 | 1.87 |
| Doughnuts | 3.64 | 1.92 |

Values with the same letters are not significantly different at the p<.05 level

**Appendix B – Main Study**

**Table S5: Secondary outcome measures for main study**

| Measure | Question | Scale |
| --- | --- | --- |
| Negative Emotional Arousal**†** | How afraid does this image make you feel?  How worried does this image make you feel?  How uncomfortable does this image make you feel?  How disgusted does this image make you feel? | 1 = not at all; 7 = very |
|  |  | 1 = not at all; 7 = very |
|  |  | 1 = not at all; 7 = very |
|  |  | 1 = not at all; 7 = very |
|  |  |  |
| Perceived Risks**‡** | Drinking this beverage often would l increase your child’s risk of heart disease  Drinking this beverage often would l increase your child’s risk of diabetes  Drinking this beverage often would l help your child live a healthier life (reverse scored) | 1 = strongly disagree; - 7=strongly agree  1 = strongly disagree; - 7=strongly agree  1 = strongly disagree; - 7=strongly agree |
| Acceptability | Would you favour or oppose a government policy requiring the above safety label to be placed on drinks? | 1 = strongly oppose; 7 = strongly support |

**†**Aggregate measure of four items measuring fear, worry, disgust, discomfort; Cronbach’s α = 0.95

**‡**Aggregate measure of four items measuring perceived risks of SSB on child’s potential to gain weight, develop hearth disease, develop diabetes and lead healthy life (reverse scored); Cronbach’s α =0.75

**Table S6:** **Regression coefficients (95% CIs) for each secondary outcome in each group against all others (pairwise contrasts conducted by varying which group was the reference group)**

| **Measure** | **Reference group** | **Control** | **Calorie information** | **Disease image label** | **Disease image & Calories information label** | **Sugar content image label** | **Sugar content image & Calorie information label** |
| --- | --- | --- | --- | --- | --- | --- | --- |
| **Negative Emotional Arousal** | **Control** |  | 0.516  (0.252, 0.779)* | 2.567  (2.305, 2.829)* | 2.273  (2.008, 2.537)* | 2.202  (1.939, 2.465)* | 2.040  (1.777, 2.302)* |
|  | **Calorie information** | -0.516  (-0.779, -0.252)* | - | 2.051  (1.790, 2.313)* | 1.757  (1.493, 2.021)* | 1.687  (1.424, 1.949)* | 1.524  (1.262, 1.786)* |
|  | **Disease image label** | -2.567  (-2.829, -2.305)* | -2.051  (-2.313, -1.790)* | - | -0.293  (-0.557, -0.032)* | -0.365  (-0.625, -0.104)* | -0.525  (-0.788, -0.267)* |
|  | **Disease image & Calories information label** | -2.273  (-2.537, -2.008)* | -1.757  (-2.021, -1.493)* | 0.294  (0.032, 0.557)* | - | -0.070  (-0.333, 0.193) | -0.233  (-0.496, 0.030) |
|  | **Sugar content image label** | -2.202  (-2.465, -1.939)* | -1.687  (-1.949, -1.424)* | 0.365  (0.104, 0.625)* | 0.070  (-0.193, 0.333) | - | -0.163  (-0.424, 0.098) |
|  | **Sugar content image & Calories information label** | -2.040  (-2.302, -1.777)* | -1.524  (-1.786, -1.262)* | 0.525  (0.267, 0.788)* | 0.233  (-0.030, 0.496) | 0.163  (-0.098, 0.424) |  |
| **Acceptabilit**y | **Control** |  | 2.407  (2.154, 2.659)* | 2.021  (1.769, 2.272)* | 2.227  (1.972, 2.481)* | 2.647  (2.394, 2.899)* | 2.849  (2.596, 3.101)* |
|  | **Calorie information** | -2.406  (-2.658, -2.153) | - | -0.386  (-0.0637, -0.134)* | -0.180  (-0.432, 0.072) | 0.241  (-0.011, 0.493) | 0.442  (0.190, 0.693)* |
|  | **Disease image label** | -2.021  (-2.272, -1.769)* | 0.386  (0.134, 0.637)* | - | 0.206  (-0.046, 0.458) | 0.626  (0.374, 0.877)* | 0.828  (0.576, 1.079)* |
|  | **Disease image & Calories information label** | -2.169 (-2.421, -1.916)* | 0.198 (-0.054, 0.450) | -0.202 (-0.453, 0.049) | - | 0.442 (0.190, 0.694)* | 0.651 (0.399, 0.902)* |
|  | **Sugar content image label** | -2.646 (-2.898, -2.393) | -0.241 (-0.492, 0.010) | -0.627 (-0.878, -0.375)* | -0.420 (-0.672, -0.167)* | - | -0.202 (-0.453, 0.049) |
|  | **Sugar content image & Calorie information label** | -2.848 (-3.100, -2.595)* | -0.442 (-0.693, -0.190)* | -0.828 (-1.079, -0.576)* | -0.622 (-0.874, -0.369)* | 0.202 (-0.453, 0.049) | - |
| **Perceived risks** | **Control** |  | -0.037 (-0.180, 0.106) | 0.142 (0.000, 0.283)* | 0.056 (-0.087, 0.199) | 0.173 (0.030, 0.317)* | 0.178 (0.034. 0.321)* |
|  | **Calorie information** | 0.037 (-0.106, 0.180) | - | 0.179 (0.037, 0.320)* | 0.093 (-0.050, 0.236) | 2.11 (1.966, 2.253)* | 0.215 (0.073, 0.356)* |
|  | **Disease image label** | -0.142 (-0.283, -0.001)* | -0.179 (-0.320, -0.037)* | - | -0.086 (-0.229, 0.057) | 0.032 (-0.109, 0.173) | 0.036 (-0.105, 0.177) |
|  | **Disease image & Calorie information label** | -0.056 (-0.199, 0.087) | -0.093 (-0.236, 0.050) | 0.086 (-0.057, 0.229) | - | 0.118 (-0.057, 0.229) | 0.122 (-0.021, 0.265) |
|  | **Sugar content image label** | -0.174 (-0.317, -0.030)* | -0.211 (-0.354, -0.067)* | -0.032 (-0.173, 0.109) | -0.118 (-0.261, 0.025) | - | 0.004 (-0.137, 0.145) |
|  | **Sugar content image & Calorie information label** | -0.178 (-0.321, -0.034)* | -0.215 (-0.356, -0.073)* | -0.036 (-0.177, 0.105) | -0.122 (-0.265, 0.021) | -0.004 (-0.145, 0.137) | - |

*Significant at the < 0.05 level.

1. Kees, J., Burton, S., Andrews, J. C., & Kozup, J. (2006). Tests of graphic visuals and cigarette package warning combinations: implications for the framework convention on tobacco control. *Journal of Public Policy & Marketing*, *25*(2), 212-223. [↑](#footnote-ref-1)
